# Assessing the Effectiveness of Histaglobulin in Chronic Idiopathic Urticaria: A Retrospective Analysis at a Tertiary Care Hospital

**DOI:** 10.7759/cureus.75519

**Published:** 2024-12-11

**Authors:** Jaskanwal Kaur, Nehal Pandya, Bhavesh Songara, Vaishali S Makwana, Dhairya Parmar, Trupti Taral

**Affiliations:** 1 Dermatology, C.U. Shah Medical College and Hospital, Surendranagar, IND

**Keywords:** chronic idiopathic urticaria, chronic spontaneous urticaria, histaglobulin, urticaria, urticaria activity score

## Abstract

Introduction

Chronic urticaria is a transient cutaneous disorder that waxes and wanes swiftly but, due to its periodic episodes, declines the quality of life of the affected individuals. It is of two types: chronic spontaneous or idiopathic and chronic-induced urticaria. Urticaria can have many different causes, but one of the most common causes of chronic idiopathic urticaria (CIU) is autoimmune. Subcutaneous injection of histaglobulin has been used for a few decades to pacify various allergic reactions. We have been using histaglobulin injection for treatment and curtailment of chronic urticaria effectively for the last two years, in addition to oral antihistamines.

Objective

This study aims to analyze the effect of treatment with injection of histaglobulin in patients of CIU from the data recorded in the Dermatology Outpatient Department (OPD) of C.U. Shah Medical College and Hospital, Surendranagar, Gujarat (CUSMC).

Methods

This is an observational retrospective review of patients with CIU treated with weekly histaglobulin injections coming to the Dermatology Department CUSMC from April 2022 to April 2024. The recorded data of 45 patients were assessed with more than six weeks of urticaria. Histglobulin injection was given subcutaneously near the deltoid region weekly for eight weeks. The Urticaria Activity Score (UAS) was recorded at each visit. A complete blood count (CBC) and serum immunoglobulin E (IgE) were done to rule out infection and allergic diathesis. The patient's history was taken to rule out other causes of urticaria. An oral non-sedative antihistaminic and soothing lotion (calamine lotion) was given to alleviate symptoms.

Results

Out of the 45 patients who underwent treatment with eight weekly injections, 28 completed therapy, 10 patients visited five to six times, and seven patients dropped treatment after two to three visits due to variable reasons. The UAS gradually decreased to lower levels after sequential injections of histaglobulin in all the patients who continued and completed the treatment protocol.

Conclusion

Our study shows a reduction of UAS in patients taking weekly histaglobulin injections. Histoglobulin significantly benefited patients suffering from the distress associated with urticaria. They had reduced dependence on oral antihistamines, and the frequency of visits to the hospital diminished.

## Introduction

Chronic urticaria is constituted by the phenomenon of wheals and itching daily or most days for more than six weeks. There are two further types of urticaria: chronic spontaneous (also called chronic idiopathic) and chronic-induced urticaria. The latter is seen in younger adults and is associated with underlying stimuli such as temperature (heat or cold), pressure, vibration, and solar due to ultraviolet A (UVA) or visible light [1]. When no association is found with a provoking factor, it is called chronic idiopathic urticaria (CIU). The course of this disease is inconsistent and may keep recurring for years. The lesions present in this condition are evanescent and pruritic, which disappear within 24 hours. It creeps in insidiously and perturbs the daily ventures of affected humans, especially in the economically productive age group, and affects learning in students [2].

The prevalence of CIU is seen across 0.1-3% of the population worldwide [3]. A major proportion of urticaria patients are due to CIU, while one-third are associated with causative factors such as underlying infections, drug-induced, pressure, solar, aquagenic, and cold urticaria. Chronic urticaria has unpredictable occurrences, which affect the mental and social health of the persons concerned. It is also an economic setback for those affected.

It is challenging to treat CIU. According to the guidelines, second-generation non-sedating H1-antihistamines are the universally recommended first-line therapy for CIU due to their efficacy in double-blind clinical studies [4]. These second-generation antihistamines are levocetirizine, fexofenadine, bilastine, olopatadine, and rupatadine. In case of non-response, the dose may be escalated from twice to fourfold. In unresponsive patients, second-line therapy is considered based on affordability and side effects. Omalizumab has been approved for chronic urticaria, which is available at a higher price. Considering that cyclosporine is available at a lesser cost for patients having poor responses to antihistamines. Methotrexate, dapsone, and autologous serum therapy are other alternative therapies. Long-term corticosteroids are not given in chronic urticaria due to their adverse effects [5].

Histopathology characteristically shows epidermal and dermal edema due to capillary dilatation in the upper and mid-dermis. Inflammatory infiltrates of neutrophils, eosinophils, lymphocytes, and mast cells are concentrated in the perivascular region. The endothelial cells are also activated in the non-lesional skin of the urticarial patients [2]. Urticaria is specifically associated with mast cells, and its degranulation is implicated where wheals are present. CIU causes itching, wheals, and angioedema by activating mast cells in the skin, which results in the production of histamine and other inflammatory mediators such as platelet-activating factor (PAF) and cytokines. This degranulation increases capillary permeability, activates sensory nerves, and promotes vasodilation, which leads to plasma leakage and cell recruitment to the afflicted areas. CIU may be caused by a variety of reasons, such as autoimmune antibodies, infections, and physical stressors, yet the exact signals that activate mast cells in CIU remain unknown. Additionally, the affected skin has increased levels of endothelial cell adhesion molecules, neuropeptides, growth factors, inflammatory infiltrates, and T lymphocytes [5]. Thus, histologically, CIU shows perivascular infiltration of monocytes, polymorphonuclear cells, eosinophils, and basophils, as well as enhanced adhesion molecules and eosinophilic activation products, indicating endothelial cell activity. Mast cells and activated endothelial cells contribute chemokines and cytokines such as IL4, IL1, and TNF-α, along with histamine and leukotrienes [6].

There are a range of treatment options available, but remission is difficult to achieve. Oral antihistamines are the preferred treatment for all types of urticaria. In cases of severity, systemic steroids are advised but can be given only for the short term due to associated side effects. Eventually, when the patient does not respond to the above therapies, immunomodulator drugs such as cyclosporine are given, which have adverse effects like hypertension and renal adversity. Plasmapheresis, methotrexate, and intravenous immunoglobulin have been tried in a few cases with little evidence [5], which have toxic effects. Autologous serum therapy (AST) is effective and has been in use for a long time, but it is more effective in autologous serum skin test (ASST)-positive individuals [7]. It is a time-consuming procedure subject to equipment and expertise [8].

Histaglobulin has been in use for a few decades in patients refractory to all other treatments of CIU [9]. It stimulates antibody formation to histamine that offers protection for six to 18 months against histamine released endogenously [10]. This immunotherapy has been suggested to be more effective than AST [8]. Histaglobulin has been tried successfully in other conditions, such as allergic rhinitis, with no side effects [11]. It has also given positive results in asthma, erythema multiforme, and drug allergies involving the skin [12,13]. Histaglobulin has been considered to decrease IgE levels while increasing serum binding capacities to histamine. In healthy individuals, 20-30% histamine binds to IgE compared to 0-5% in allergic individuals [14]. It is also a pocket-friendly treatment, as weekly single shots are given subcutaneously.

## Materials and methods

This two-year retrospective study utilized data from patients with urticaria in Dermatology OPD who had symptoms for more than six weeks and were diagnosed at C.U. Shah Medical College and Hospital, Surendranagar, Gujarat (CUSMC). The patients included in the study were refractory to other forms of treatment.* *Patients who were diagnosed with CIU were given the first and second lines of treatment in the form of antihistamines such as levocetirizine in standard dosage, double dosage, and in combination with bilastine for two to three months. Drugs such as cyclosporine and omalizumab were not given due to the side effects and cost factor in this setting. The sufferers who did not respond were given intermittent subcutaneous injections of histaglobulin weekly. The record obtained was from April 2022 to April 2024. The inclusion criteria for the study included all patients above the age of 15 who were diagnosed with CIU and coming to the Dermatology OPD as per the record available. Pregnant females and immunocompromised individuals were excluded from this study.

The patients were subjected to subcutaneous 1 mL histaglobulin weekly for eight weeks. They continued with a daily dose of oral antihistaminic tablet levocetirizine 10 mg in once daily (OD) and twice daily (BD) dosage, tablet fexofenadine 60-180 mg, and bilastine 20-40 mg. Injection histaglobulin is an innovative product of Bharat Serum and Vaccines Limited Maharashtra (Navi Mumbai, India). It is a combination of normal human immunoglobulin (12 mg) and histamine dihydrochloride (0.15 mg), a sterile preparation of histamine fused to active protein gamma globulin from human blood. The manufacturers claim to test the product negative for hepatitis B Ag, human immunodeficiency virus (HIV) 1 and 2, and hepatitis C virus (HCV) RNA by polymerase chain reaction (PCR) [15]. The mechanism of action of histaglobulin has not been unraveled yet.

The Urticaria Activity Score over seven days (UAS7) [14] was used to assess the feedback from the patients by four parameters: wheal, erythema, pruritus, and angioedema based on the number, size, and duration. A score of 0 denotes no urticaria. A score of 1 indicates mild severity and includes up to 20 wheals; a moderate score of 2 has 20-50 wheals causing discomfort to the patient; and a score of 3 has an intense severity of more than 50 wheals. The daily score ranges from 0 to 6, with a maximum of 42 per week. The number was recorded and assessed weekly.

## Results

**Figure 1:**
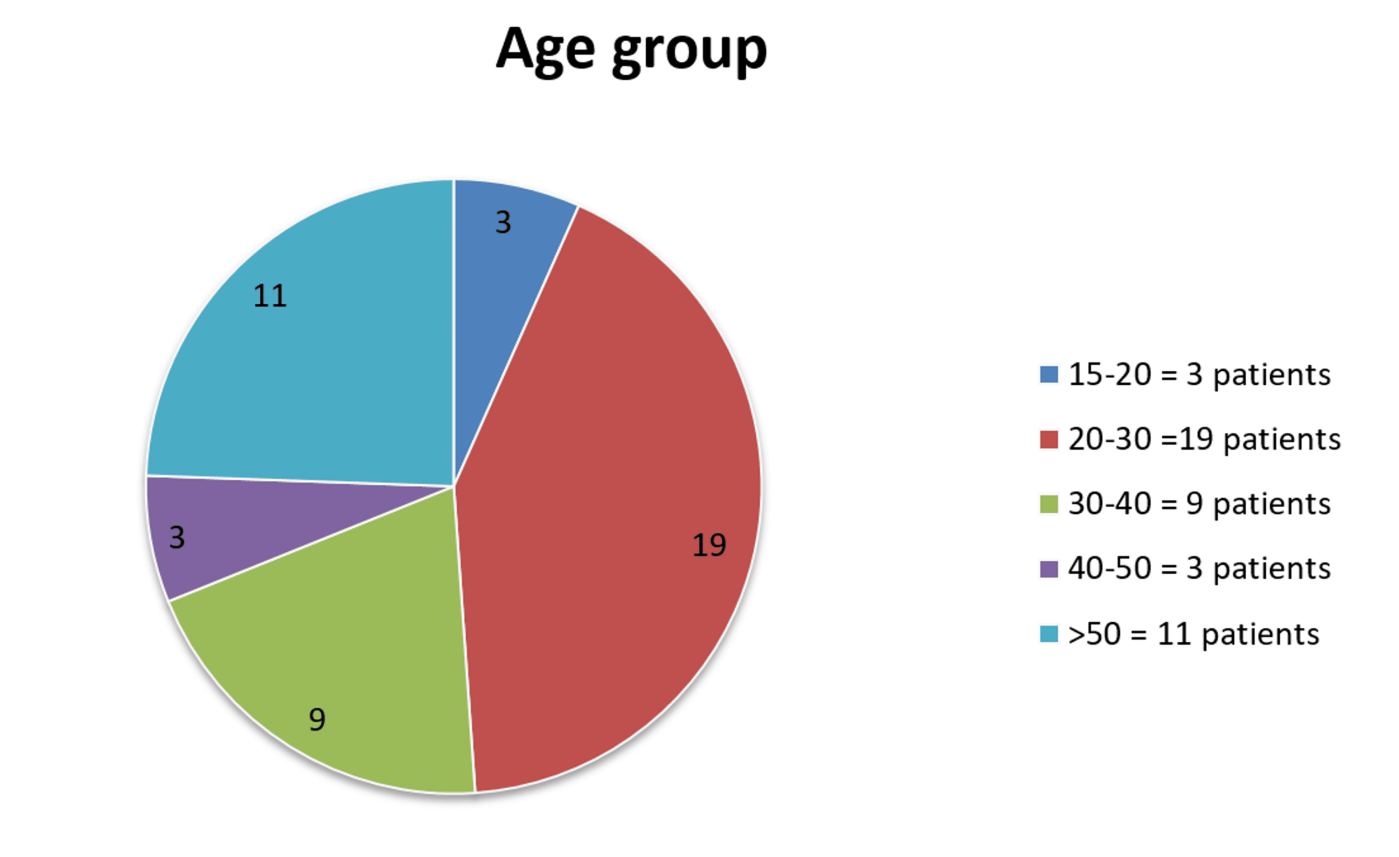
The age range of the patients.

**Figure 2:**
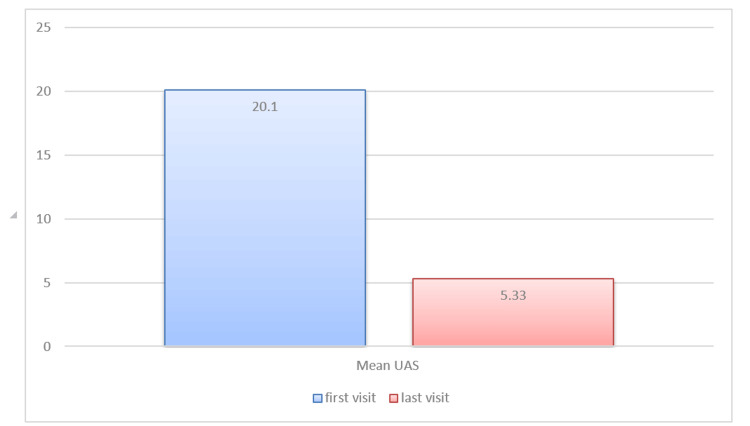
The UAS at the first versus the last visit for the mean of patient scores. UAS: Urticaria Activity Score

**Figure 3:**
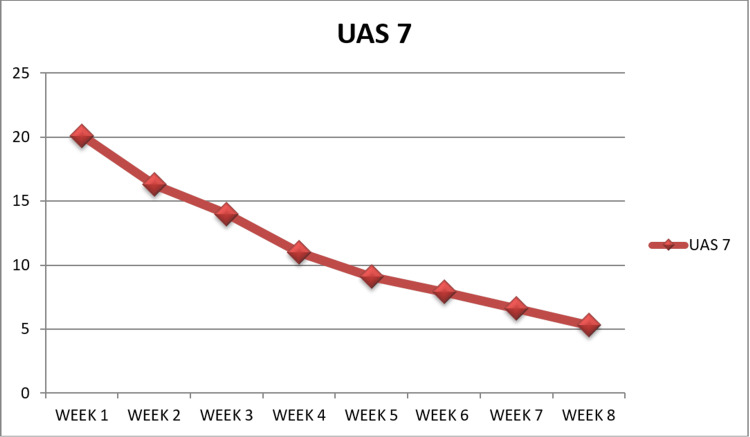
The treatment response of histaglobulin injections in patients as measured by the UAS7. UAS: Urticaria Activity Score

In this study, the patients' UAS points were monitored over a period of eight weeks while receiving treatment. The mean age of patients was 29.4 years (Figure 1). The majority of patients belonged to the 20-30 age group, with 19 patients; 11 were above 50 years of age, and three were younger, falling between 15-20 years. Among the 45 patients treated, 25 were males and 20 were females (Figure 2), contradicting the finding of outnumbered females in CIU [16]. The mean basal UAS at the start of treatment was 20.1, which was later drastically reduced to 5.33 after the completion of treatment, showing a 73.6% decrease in UAS by the eighth week (Figure 3). After eight weeks, complete remission occurred in five patients, while 15 patients showed a significant response. The mean UAS reduced to 10 in 10 patients, but most of the patients did not come for repeat injections after a significant drop in the UAS. Adverse effects were not reported in any of the patients other than mild pain at the injection site.

## Discussion

Chronic urticaria is a perpetual, incessant disorder that is mentally and physically taxing for the patients. Ordinarily, mast cells, basophils, and eosinophils are the underlying cells involved in urticaria. The chemical molecules such as histamine, leukotrienes, and substance P unfold the events of urticaria. Mast cells, which are predominantly involved in chronic urticaria, trigger provocation of sensory nerves along with vasodilatation, leading to leakage of inflammatory cells such as basophils, eosinophils, and T cells [4,5]. The endothelial cells eventually lead to wheals, itching, and urticaria [6]. The IgE receptors on mast cells facilitate the degranulation of mast cells, which leads to the events of chronic urticaria. The main causative factor is histamine, which causes cutaneous vascular dilatation and serum leakage. This mechanism, when paired with C-fiber activation, causes itching. Leukotrienes, basophils, and eosinophils have similar effects and prolong the episode of urticaria. Newly synthesized leukotrienes have been shown to promote inflammation in chronic urticaria. While not implicated in early wheal formation, basophils exacerbate lesions through migration and histamine release. Eosinophil activation and infiltration are seen in histological studies of chronic urticaria lesions [17]. Genetic association of *HLADR4* has been found to exist in some cases of chronic urticaria.

Antihistaminics, especially nonsedatives, have been constantly used to provide symptomatic relief, but the disease response is unpredictable. UAS primarily focuses on the presence of itch and the number of wheals, a daily score that ranges from 0 to 6 using the Likert-type symptom intensity scale 0-3 (Table 1) [11]. Both routine clinical practice and several controlled clinical trials have made use of the UAS. It was just verified, especially for the purpose of tracking and monitoring chronic urticaria activity, and it was advised to be used for at least four days in a row, if not for a week (UAS7). UAS is assessed by wheals in totality and the vigor of itching with it on a scale of 0-3. This is determined daily with a maximum of six points per day and 42 per week [2].

**Table 1:** The UAS points from week one to week eight, representing the mean for all the patients. UAS: Urticaria Activity Score

Week	Mean UAS	Number of Patients
1st week	20.1	45
2nd week	16.2	38
3rd week	14	34
4th week	11	32
5th week	9.2	27
6th week	7.9	21
7th week	6.6	19
8th week	5.3	12

Subcutaneous histaglobulin injection triggers the body to develop antibodies to histamine, which are protective against endogenous histamine released by the body due to allergies [18]. Repetitive administration of histaglobulin increases the antibody levels, and cyclic dosage keeps sufficient antibody titers [13]. Histaglobulin lowers IgE levels, and it is thought to boost the patient's ability to bind histamine in this serum [19]. Of late, histamine has been a preferred treatment for allergic rhinitis and other allergic conditions such as asthma, allergic dermatitis, erythema multiforme, cutaneous drug allergy, and CIU [12,19]. Rudzki and Czubalski (1967) were the first to throw light on the effectiveness of histaglobulin in CIU as well as prurigo [20]. Gushchin et al. also showed a similar ability of histaglobulin in chronic recurrent (idiopathic) urticaria along with allergic rhinitis [14].

The limitations of the study include the reliance on available records, which restricted the control aspect of the study. Additionally, ASST was not performed on the patients, and detailed medical histories were not available.

## Conclusions

The study shows histaglobulin as an effective therapeutic mode of treatment in CIU. There was a threefold decrease in the mean UAS by the eighth week as the score reduced from 20.1 to 5.3. The requirement of antihistamines was also reduced. The patients who achieved remission before eight weeks stopped coming for follow-up visits after a decrease in the UAS. This signified that the therapeutic effect of histaglobulin was earlier in some patients, while few required longer treatment. No adverse effects were observed in the patients other than mild pain at the injection site. We can consider histaglobulin to be a new, effective, economical therapeutic modality of treatment.
